# Functional and Traditional Resistance Training Are Equally Effective in Increasing Upper and Lower Limb Muscular Endurance and Performance Variables in Untrained Young Men

**DOI:** 10.3389/fphys.2022.868195

**Published:** 2022-08-31

**Authors:** Chongwen Zuo, Shumin Bo, Tao Wang, Wei Zhang

**Affiliations:** ^1^ Graduate Department of Capital University of Physical Education and Sports, Beijing, China; ^2^ School of Kinesiology and Health of Capital University of Physical Education and Sports, Beijing, China; ^3^ School of Physical Education, Liaocheng University, Liaocheng, China; ^4^ College of Basic Medical, Jining Medical University, Jining, China

**Keywords:** functional resistance training, traditional resistance training, physical performance, muscle strength, muscular endurance

## Abstract

**Background:** Functional resistance training (FRT) has been proposed as a safe alternative to traditional resistance training (TRT) for developing neuromuscular adaptation capacity and improving muscular strength and competitive performance. This study sought to compare the effects of 6 weeks of FRT and TRT on upper and lower limb muscular endurance and performance variables in untrained young men.

**Methods:** Twenty-nine untrained healthy young males aged 18–29 years were randomly given 6 weeks of FRT [40% of 1repetition maximum (RM), 4,5 sets of 20 repetitions, 3 times/week] or TRT (70% of 1RM, 4,5 sets of 12 repetitions, 3 times/week). All participants underwent numerous tests before and after the 6-week training, such as muscular endurance (reps of bench press and leg flexion) and physical performance tests (sprint performance, pull-ups, throwing ability, and jumping ability).

**Results:** After the 6 weeks of training, the TRT and FRT groups showed an equally significant increase in muscular endurance (*p* < 0.01), while the throwing and jumping abilities, 30-m sprint, and pull-ups performances in both the groups (*p* < 0.01) also improved significantly. However, no differences were observed between the groups (*p* > 0.05).

**Conclusion:** These findings indicate that both functional resistance training and traditional resistance training are effective training methods for improving the upper and lower limb muscular endurance and performance in untrained young men.

## Background

Previously used for treating functional and partial deterioration in old adults as well as stroke patients (Scholtes et al., 2012; Lee et al., 2013) and postoperative rehabilitation patients (Ageberg et al., 2008), functional resistance training (FRT) on unstable surfaces (e.g., BOSU ball, Swiss ball, and balance disc), is now employed as a new training technique for improving sports performances (Thompson, 2021). Conceptually, FRT is a set of exercises performed to enhance performance in daily functions (Fowles, 2010) or develop the ability to perform activities of daily living (Thompson, 2016). It features several dynamic exercises containing synchronized, multidimensional, and numerous joint movements conducted on unstable surfaces for developing different physical conditioning (e.g., muscle strength) and performance variables (e.g., power and speed) for increased core stability (La Scala Teixeira et al., 2017; Feito et al., 2018). It is suggested that FRT should focus more on improving movement patterns rather than concentrating on specific muscular adaptations, as done in another fitness-enhancing exercise, traditional resistance training (TRT). It was reported that regular resistance training imparted more attention to specific muscles for enhancing strength and physical performance by gradually increasing the training load in either fixed or stable positions (Tomljanović et al., 2011; Feito et al., 2018).

Previous studies have reported the effects of FRT were generally observed in athletes, older adults, and diseased patients and seldom covered healthy untrained young individuals. For example, a systematic review (Xiao et al., 2021) concluded that although FRT significantly improved athletes’ muscular strength, power, speed, and agility, no significant effects were found in muscular endurance and anthropometric variables. Another study by (Bale and Strand, 2008) reported that a 4-week FRT of the lower limbs gave better results than TRT in promoting functional performance and muscular strength in 18 post-stroke patients in the subacute phase. Similarly (Abbaspoor et al., 2020), also indicated that an 8-week combined FRT might be an effective training model for increasing the walking speed, quadriceps, and handgrip strength in women with multiple sclerosis (MS). Tomljanović et al. (2011) studied healthy young kinesiology students during a 5-weeks program and demonstrated that while FRT improved postural control and coordination, TRT augmented the energetic potential of trained musculature; thus, increasing the strength. Several studies conducted on inexperienced healthy individuals suggested that instability resistance training that engages lower forces can improve maximal strength (Milovan et al., 2012), power (Mate-Munoz et al., 2014), movement velocity, and jumping ability (Sparkes and Behm, 2010) similar to TRT held under stable conditions along with heavier loads. However, these studies only involved the effects of local exercise on physical capacities. There are no studies to date that have compared the instability resistance training with TRT programs in untrained young men in terms of muscular endurance and performance for several weeks. This might be significant in cases of two training programs having a similar training volume; however, there is little empirical data to suggest that FRT can greatly improve muscular endurance.

Several studies have reported that similar exercises, when performed under unstable parameters as compared to stable conditions (e.g., muscular strength, power, and speed), display increased physical capacities (Tomljanović et al., 2011; Yildiz et al., 2019; Xiao et al., 2021), as physical capacities play a crucial role in determining players’ competitiveness. However, Behm et al. (2010) stated that unstable devices are not always effective in meeting the specific demands (e.g., strength and balance) of the athletes. For example, if an athlete needs to develop optimal strength and power, then training under unstable surfaces that require reduced external load and force is not very efficacious for trained athletes (Behm and Colado, 2012; Behm et al., 2015; La Scala Teixeira et al., 2017). On the contrary, unstable resistance training can also be employed by an untrained population to improve strength and power and promote functional health benefits. It is reported that resistance training under unstable conditions might impart instability in the performance of daily activities, occupations, and sports, thus, providing more beneficial training adaptions and transfer (Tomljanović et al., 2011). Another study by Behm and Colado (2012) reported that a 30% force deficit induced by unstable resistance training can be beneficial, as the lower load and torque might reduce the risk of training injuries or improve the functional restoration after injury. However, a meta-analysis suggested that balance training is highly task-specific in trained and untrained individuals (Kummel et al., 2016). Furthermore, the effects of resistance training on unstable surfaces are inconsistent and unfavorable for developing muscular fitness, especially muscular strength, when compared with stable conditions (Behm et al., 2015). Thus, to our knowledge, no study to date has compared the effects of two types of equal-volume resistance training schedules (functional vs. traditional) on upper and lower limb muscular endurance and performance in untrained young men.

Therefore, this study aimed to compare the distinct effects of FRT and TRT protocols, having equal training volume, on upper and lower limb muscular endurance and certain specific performance variables (e.g., sprint performance, pull-up, throwing ability, and jumping ability) in untrained men for over 6 weeks. Our hypothesis suggests that both groups would show a significant increase in all performance indicators, although the FRT group might display greater muscular endurance enhancements.

## Materials and Methods

### General Design

This study was designed as a randomized controlled trial and was prospectively registered at the http://www.chictr.org.cn/as ChiCTR2100048485, with ethical approval granted by the Capital University of Physical Education and Sports ethical committee. Before study initiation, all the participants were informed of the risks and requirements of the training program, and voluntary consent was obtained from all of them. This paper followed the CONSORT statement (Schulz et al., 2010).

### Participants

**FIGURE 1:**
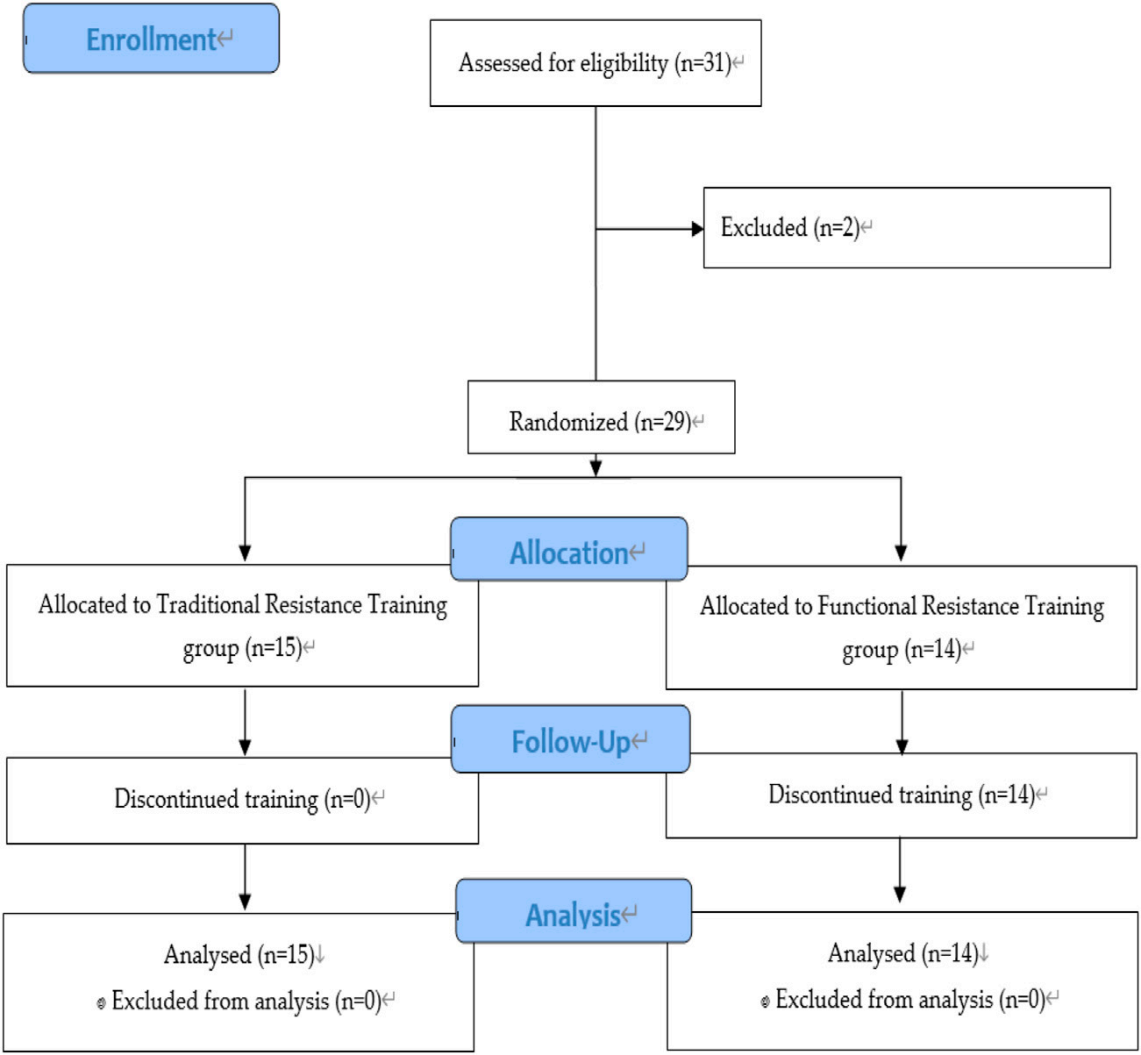
Study Flow chart (Schulz et al., 2010).

A total of 31 untrained individuals were initially screened at the Capital University of Physical Education and Sports in Haidian District, Beijing, China (Figure 1). All the participants were recruited through print and word-of-mouth advertising. The inclusion criteria were as follows: 1) participants ≥18 years old, 2) they did not undergo any regular resistance-type training for 6 months before the study commenced, and 3) patients did not regularly smoke, drink alcohol, or consume any medications, 4) patients without overt chronic diseases and sports injury. Consequently, 29 participants met the inclusion criteria, while two were dropped out because of personal reasons. All the participants were randomly assigned to either the TRT (*n* = 15) or the FRT groups (*n* = 14) and were instructed not to attend any extra training and to maintain normal eating habits throughout the 6-week training period.

### Anthropometric Measurements

The height and weight were measured using a portable stadiometer and an electronic scale before and after a 6-week regular resistance training intervention. Then, the body mass index (BMI) was calculated according to the following formula: BMI = weight (kg)/height (m)^2^. Anthropometric measurements of the participants who fasted overnight (>8 h) were simultaneously assessed before and after the resistance training intervention.

### Test Procedures

All participants performed the assessment process before, during, and after the 6-week intervention. The test procedure involved two separate phases with a gap of 24 h. The first testing day included anthropometric measurements and a 1RM test, including barbell squat, bench press, deadlift, and right leg flexion. The second phase incorporated throwing and jumping abilities, sprint achievement, pull-ups, and muscular endurance tests. In order to avoid the influence of the muscular endurance test on other test results, the upper and lower limb muscular endurance measurements were arranged as the last measurement of all assessments. Participants were asked not to undergo any physical exercise a day before and avoid taking food, caffeine, and alcohol 12 h before the measurement.

### Maximal Strength Measurements

Each participant completed the 1RM test before the 6 weeks training program in the same order, i.e., barbell squat, bench press, deadlift, and seated leg flexion. The 1RM tests conformed to the prescribed guidelines of the American College of Sports Medicine (American College of Sports Medicine et al., 2018). Measurements were taken by gradually increasing the weight lifted by the participants until they failed to lift the current weight throughout the exercise. Initially, the participants performed a 5 min warm-up on a paddle ergometer at a perceived exertion level of 3 (on the CR 10 Borg scale), followed by two warm-up sets of 5–10 repetitions at 40–60% 1RM. For the last set, participants performed three to five repetitions at approximately 60–80% 1RM, while a 1–2 min rest period was allowed between the warm-up sets. After the last set, a 3 min rest was taken before the actual 1RM test. Participants completed the test in five trials, with a rest period between each trial set of approximately 3 min, and the highest load achieved was recorded as the 1RM load. Before each strength test, participants were instructed to understand each test movement pattern, especially the bench press, which required the participants to lower the bar to the chest without touching as well as keeping the upper arms parallel to the ground followed by returning the bar upward and successfully straightening the elbow at “press” command. Barbell squats were performed while the participants held a bar on the back and core fully stretched perpendicularly to the knee. All participants were further asked to keep their feet shoulder-width apart at a 45° angle throughout the test. As the participants were in a seated position, the hip angle was approximately 110° in the leg flexion test. With verbal encouragement, the participants attempted to perform a concentric dominant leg flexion starting from the extended position at 180° to reach an approximate flexion of 70° against the resistance loads (kg).

### Muscular Endurance Measurements

Upper and lower limb muscular endurance were assessed by bench press and leg flexion tests; participants were instructed to complete the maximum number of repetitions (reps) of bench press and leg flexion, respectively. The same load (70% of 1RM) was used for pre-and post-intervention measurements as suggested by a previous study (Hackett et al., 2021). According to the 1RM test, participants were asked to achieve a full range of motions and proper techniques. The repetition cadence was performed in 1 s eccentric and concentric contractions. The maximum number of reps and the volume load for each exercise were recorded for statistical analysis.

### Physical Performance Measurements

Physical performance measurements consisted of throwing ability, jumping ability, 30-m sprint, and pull-ups. To assess the throwing ability, a medicine ball throw (MBT) test was used in which participants were kept behind a line marked on the floor in a seated position and were instructed to sit on the floor with their head, shoulder, and back against the wall. Their legs were straight apart and facing the direction in which the ball was thrown. A 2 kg medicine ball was held in their hands with arms at 90° to the shoulder abduction, similar to a chest pass in basketball, and they were told to throw the ball horizontally. Additionally, participants were also further instructed not to use their lower body for exerting force with their head, shoulder, and back pressed against the wall. Participants completed three practice trials with a 1-min rest between each trial. The average of these multiple readings was used for analysis.

The Quattro Jump System (Kistler 9290AD, Switzerland) was used to evaluate the jumping ability. All participants performed a countermovement jump (CMJ) test without swinging their arms from the portable force plate. For the starting position, the participants stood straight on the force plate with their hands on the hips, but after the instructor’s cue, they squatted down rapidly to a 90° knee angle position and jumped straight up as high as possible, with their hands on the hips. During the ascending phase, the participants left the force plate with the fully stretched lower limbs and landed on both feet on the force plate with straight knees to measure the airtime. As suggested by a previous study (Sattler et al., 2012), the best of three consecutive trials, with appropriate rest allowed between each trial, was used as the final test result.

In the 30-m sprint test, participants were asked to sprint a distance of 30 m while passing through a photocell (Brower Timing System, United States). The participants started on the sound signal, which activated the timer system. Two sets of photocells were placed at the 30-m gates. The timing results from individual gates were recorded as the result of a 30-m sprint. The best of two consecutive tests was selected as the final result for the statistical analysis.

The pull-up test was performed starting from a dead hang position with the arms fully stretched and locked and feet off the floor. The bar was clasped with hands in pronation, set apart by a distance wider than the shoulders. From this position, the entire body was lifted until the chin was higher than the bar. On the way down, the body was kept straight, hanging down from the bar with fully stretched arms. This procedure was repeated until they could not finish a pull-up, and the number of pull-ups was recorded.

### Exercise Interventions

#### TRT Protocol

Table 1 presents the summary of the TRT and FRT protocols. The participants in both groups were trained for 18 sessions (of 60 min each) thrice a week for six consecutive weeks. Each session time contained a 5–10 min warm-up on a wind ergometer before every workout, while the remaining 50 min of the session was spent in the whole-body workout. The TRT program comprised five exercises, namely barbell squat for the lower limb, horizontal bench press for chest muscles, deadlift for back and leg muscles, reverse arm curl for biceps, and seated leg flexions for quadriceps in stable conditions (70% of 1RM, and 4,5 sets of 12 repetitions), with 1,2 min of rest between the sets.

**TABLE 1:** Resistance training protocols.

Group	Exercises	Sets	Repetitions	Training Intensity	Rest
TRT	Barbell Squat	4,5	12	70%1RM	1,2 min
	Bench Press	4,5	12	70%1RM	1,2 min
	Deadlift	4,5	12	70%1RM	1,2 min
	Reverse Arm Curl	4,5	15	10 kg	1,2 min
	Leg Flexion	4,5	15	70%1RM	1,2 min
FRT	Barbell Squat & BOSU	4,5	20	40%1RM	1,2 min
	Bench Press & Swiss ball	4,5	20	40%%1RM	1,2 min
	Deadlift & BOSU	4,5	20	40%1RM	1,2 min
	Kettlebell Swing & BOSU	4,5	15	20 kg	1,2 min
	Bulgarian Split Squats & BOSU	4,5	15	16 kg	1,2 min

#### FRT Protocol

The FRT group performed the same training exercises as the TRT group on unstable devices (e.g., BOSU ball, Swiss balls, and balance discs). Moreover, an unstable training schedule may not provide the same intensity of muscle overload as TRT under stable conditions while considering safety factors (Kibele and Behm, 2009). The horizontal bench press, deadlift, and barbell squat were performed on the Swiss ball, balance disc, and BOSU ball, while kettlebell swings and Bulgarian split squats were performed on the BOSU ball, respectively. The equivalents of the total training volume were coordinated between the two groups. The repetition in the FRT group was calculated using the following formula: 70% 1RM lifting weight (kg) × reps (TRT group)/40%1RM to volition fatigue, with 1,2 min of rest between sets. Thus, the FRT group performed 4,5 sets of 20 repetitions at 40% 1RM with 1,2 min of rest between sets. The strength assessment for all participants was done again after 3 weeks of intervention to ensure that the participants had readjusted training intensities based on their strength gains. All the participants were asked to maintain normal dietary habits and avoid overeating to minimize any potential diet-induced variability in muscle strength and body composition measurements.

### Statistical Analysis

Statistical analyses were performed using SPSS version 22.0 Windows (SPSS, Inc. Chicago, IL, United States). The sample size was estimated based on a similar experimental design (Unhjem et al., 2016). Moreover, with an effect size *f* ^2^ = 0.30, a power of 0.80, and a significance level of 0.05 (Cohen, 1992), the minimum sample size of 24 (12 per group) was found to be adequate using repeated measurements analysis of variance (ANOVA, G*Power 3.1; Heinrich Heine, Dusseldorf, Germany). All baseline and post-intervention data were normally distributed utilizing the Shapiro-Wilk’s W test, which indicated appropriate normality in the distribution for all variables. All pre-and post-intervention data were expressed as mean ± standard deviation (SD). An independent sample *t*-test was used to test the pre-intervention measurement difference between the two groups. Training effects were analyzed using a mixed two-way repeated-measures ANOVA ([time (pre-and post-training)] — training group (TRT and FRT)] to verify differences in muscular endurance and physical performance between the groups. Post-hoc tests were applied using the Bonferroni corrections. The mean difference of changes in muscular endurance and physical performance for each group was presented. Furthermore, the effect sizes were calculated as partial eta square and converted to Cohen d, being classified as small (0–0.2), medium (0.2–0.8), and large (>0.8). A *p*-value < 0.05 was considered statistically significant.

## Results

### Participants

Table 2 presents the main characteristics of all the participants at baseline. No significant differences between the groups were observed in terms of age, height, body weight, body mass index, and 1RM tests. Additionally, all the participants in the groups adhered to the scheduled 18 training sessions during the intervention period. No training-related injuries, as well as participant withdrawal, were observed.

**TABLE 2:** Anthropometric characteristics of the participants at baseline.

Test	TRT (*n* = 15)	FRT (*n* = 14)	*p*-value
Age (y)	22.1 ± 2.9	20.9 ± 2.7	0.262
Height (cm)	176.6 ± 5.4	176.7 ± 6.0	0.957
Body mass (kg)	77.9 ± 11.6	73.4 ± 10.2	0.270
BMI (kg/m^2^)	24.9 ± 3.1	23.4 ± 2.6	0.168
BP (kg)	75.0 ± 9.8	71.4 ± 10.3	0.348
BS (kg)	116.0 ± 19.9	114.3 ± 16.0	0.801
DL (kg)	118.7 ± 21.3	110.0 ± 25.4	0.310
R-LF (kg)	43 ± 6.5	39.3 ± 6.8	0.143

BMI body mass index, BP bench press, BS barbell squat, DL deadlift, R-LF right leg flexion, TRT traditional resistance trainings, FRT functional resistance training.

### Muscular Endurance

Table 3 presents the results of muscular endurance tests. Both training protocols displayed increased bench press (repetitions) for the upper limb muscular endurance (TRT +10.1reps, *p* = 0.000, FTR +12.4reps, *p* = 0.000, Cohen d = 0.43), right leg flexion (repetitions) for the lower limb muscular endurance (TRT +8.1, *p* = 0.000, FTR +7.9, *p* = 0.000, Cohen d = -0.03), with a main effect of time (*p* < 0.001) and no difference between groups. Additionally, muscular endurance expressed as volume-load also significantly increased in both the groups for the bench press (TRT +508.7 kg, *p* = 0.000, FRT +587.9 kg, *p* = 0.000, Cohen d = 0.23) and the right leg flexion tests (TRT +251.3 kg, *p* = 0.000, FRT +214.8 kg, *p* = 0.000, Cohen d = -0.13) without any significant difference between the training groups.

**TABLE 3:** Change in upper and lower limbs muscular endurance as mean difference, a statistical test of group difference and effect sizes as Cohen d.

Test	Group	Pre	Mid	Post	Md	Es a	Es b	*p* ^G^
BP Rep	TRT	19.5 ± 5.5	26.7 ± 5.0##	29.7 ± 6.3**	10.1	1.84	0.43	0.374
	FRT	17.6 ± 5.3	25.1 ± 6.8##	30.0 ± 7.1**	12.4	2.34		
BP VL (kg)	TRT	1,033.2 ± 341.4	1,394.4 ± 289.0##	1,541.9 ± 330.2	508.7	1.49	0.23	0.510
	FRT	897.1 ± 361.1	1,256.9 ± 426.6##	1,484.9 ± 375.5**	587.9	1.63		
R-LF Rep	TRT	21.6 ± 5.2	25.7 ± 7.0##	29.7 ± 8.3**	8.1	1.56	−0.03	0.907
	FRT	23.3 ± 9.2	25.6 ± 6.0##	31.1 ± 7.8**	7.9	0.86		
R-LF VL (kg)	TRT	661.0 ± 207.8	787.5 ± 274.6##	912.2 ± 327.8**	251.3	1.21	−0.13	0.570
	FRT	679.0 ± 357.4	731.5 ± 232.8##	893.8 ± 304.9**	214.8	0.60		

BP bench press, R-LF right leg flexion, Rep repetition, VL volume-load, TRT traditional resistance training group, FRT functional resistance training group, MD mean difference Post-Pre, ES a effect sizes within the group as Cohens d, ES b effect sizes between groups as Cohens d, *p* G value of the difference between groups, Mid-Pre ##*p* < 0.01, Post-Pre **p < 0.01.

### Physical Performance

As shown in Table 4, a significant difference in throwing and jumping abilities was observed. The MBT performance increased by 0.4 and 0.3 m in TRT and FRT groups, while the CMJ performance increased by 6.7 and 5.0 cm in TRT and FRT groups, respectively, with no significant difference between the groups; the effect sizes indicated small effects (Cohen d = –0.18 for MBT and –0.17 for CMJ).

**TABLE 4:** Change in physical performances as mean difference, statistical test of group difference and effect sizes as Cohen d.

Test	Group	Pre	Mid	Post	Md	Es a	Es b	*p* ^G^
MBT (m)	TRT	5.9 ± 0.4	6.0 ± 0.4##	6.2 ± 0.4^**^	0.4	1.00	−0.18	0.513
	FRT	5.9 ± 0.7	6.1 ± 0.6##	6.3 ± 0.6^**^	0.3	0.43		
CMJ (cm)	TRT	59.1 ± 9.1	65.1 ± 5.0#	65.9 ± 5.2^**^	6.7	0.74	−0.17	0.483
	FRT	61.3 ± 10.7	66.2 ± 10.9##	66.3 ± 10.3^**^	5.0	0.47		
CMJ power	TRT	20.7 ± 2.9	22.4 ± 2.6##	23.4 ± 2.8^**^	2.7	0.93	0.04	0.753
	FRT	20.1 ± 3.8	21.9 ± 3.3#	23.1 ± 3.0^**^	3.0	0.79		
30 m sprint(s)	TRT	4.1 ± 0.3	3.8 ± 0.3##	3.8 ± 0.3^**^	−0.3	−1.0	0.00	0.343
	FRT	4.1 ± 0.2	3.7 ± 0.2##	3.7 ± 0.2^**^	−0.3	−1.5		
Pull-ups (reps)	TRT	8.1 ± 3.5	10.1 ± 3.7##	12.5 ± 3.7^**^	4.5	1.29	−0.07	0.303
	FRT	8.9 ± 4.0	11.1 ± 4.5##	12.9 ± 4.2^**^	4.0	1.0		

MBT medicine ball throw, CMJ countermovement jump, TRT traditional resistance training group, FRT functional resistance training group, MD mean difference Post-Pre, ES a effect sizes within the group as Cohens d, ES b effect sizes between groups as Cohens d, *p* G value of the difference between groups, Mid-Pre #*p* < 0.05 ##*p* < 0.01, Post-Pre **p < 0.01.

An improvement in 30-m sprint and pull-up performance tests was observed in both the groups; however, all the analyzed measurements were significantly different from the baseline. 30-m sprint increased by 0.3s in TRT (*p* = 0.002) and FRT groups (*p* = 0.000), respectively. Similarly, for pull-ups performance, the TRT group improved by 4.5 as compared to 4.0 in the FRT group. However, these results did not differ between the training protocols. The effect sizes indicated small effects for the 30-m sprint test (Cohen d = 0.00) and pull-ups (Cohen d = –0.07), respectively.

## Discussion

The present study was designed to compare the effects of the 6-week supervised TRT and FRT protocols with equal volume on upper and lower limb muscular endurance and physical performance in untrained healthy men. Our results suggested that both resistance training modalities (functional and traditional resistance training) produced similar training effects in untrained healthy young men over a 6-week intervention period. No pre-to post-test significant differences were detected in the training-induced improvements in parameters such as repetitions and volume-load in the bench press, leg flexion, MBT distance, CMJ height, 30-m sprint time, and pull-ups. In a study, Sparkes and Behm (2010) reported that unstable resistance training had a tendency for a smaller instability-induced force deficit in comparison with the force produced with the stable training. However, no difference between TRT and FRT groups was found during the muscular endurance and performance assessment in our study. Therefore, it is stated that unstable resistance training is also an effective method for developing force during a brief training period (Sparkes and Behm, 2010).

However, contrary to our hypothesis, the muscular endurance enhancement in the FRT group was not significantly greater than in the TRT group. It was discovered that an increase was seen in the repetition of bench press and leg flexion, which was similar between the groups, whereas enhanced volume load was observed in both the groups after 6 weeks of training. Our results indicate that high-intensity resistance training elicited greater metabolic stress than lower-intensity resistance training; the specific stimuli provided by a traditional protocol did not translate into enhanced muscular endurance. The evidence suggests that high repetitions (≥20RM) with lighter loads are efficient in enhancing muscular endurance under equal training volume. Additionally, Campos et al. (2002) reported that no difference was observed between low, moderate, and high repetition groups with equal volume despite excellent muscular endurance observed in the high repetition group, which was in accordance with our study. Therefore, it is suggested that traditional high-intensity/instability and low-intensity resistance training might induce muscle capillarization and mitochondrial adaptation, while the enhanced muscular endurance provided by instability resistance training could also be a cumulative result of better tolerance in unstable conditions.

Our study is the first preliminary study that has investigated the FRT effects on the CMJ, as well as compared the effects of 6-week TRT and FRT protocols in untrained young men; our results indicated that both were equally beneficial in promoting the jumping height. Recent evidence states that TRT improves the jumping ability (Fatouros et al., 2000; Tomljanović et al., 2011; Yildiz et al., 2019). However, a few studies focusing on the FRT effects on vertical jumping ability demonstrated that although vertical CMJ increased after long-term FRT (Yildiz et al., 2019; Keiner et al., 2020), FRT did not have a great advantage in improving explosive force, which was contrary to a study done on non-athletes (Liu et al., 2014). By contrast, the results of another two studies showed that FRT protocol did not improve jumping abilities (Cressey et al., 2007; Tomljanovic et al., 2011), which was inconsistent with our study. Additionally, two main reasons explaining the inability of Cressey’s and Tomljanović’s protocols to improve participants’ jumping abilities were elucidated. Firstly, their FRT protocol mainly performed upper limb/lower limb exercise, whereas there were five exercises covering the main muscle groups of the whole body in our protocol design, which is the biggest difference from their exercise protocols design. Secondly, since their participants were trained men, the training stimulation might not have affected them to the same degree as the untrained young men. For the reasons mentioned above, our study results were inconsistent with findings from the previous studies. In addition, we speculated that TRT and FRT protocols seem to increase the force generated by joints, which might lead to some improvement in the measured jumping ability.

Explosive strength or performance was influenced by several dominant factors, that included force generated by joints, muscle force development rate/muscle power, and neural coordination of movement (Tomljanović et al., 2011). Considering that the FRT protocol of this study covered main muscle groups of body, the FRT group obtained enough training stimulation for explosive strength performance, which significantly improved their throwing ability. Moreover, we deduced that the improvement in throwing is mainly connected with neuromuscular coordination. It is due to the fact that the training imparted using unstable devices in which most emphasis is placed on trunk region control and muscular coordination. It was found that multiple joints participated in movements during the MBT test, either in eccentric-concentric contractions of the shoulders and trunk regions, or to ensure stability of the non-active parts of the hip and lower body regions (Tomljanović et al., 2011), the significant improvement in throwing ability by our FRT protocol seemed to be logical.

Regarding other physical performance tests, we observed a significant improvement in 30-m sprint and pull-ups from baseline, and no difference was noted between the groups; therefore, both the TRT and FRT protocols were effective training methods in improving the performance of 30-m sprint and pull-ups in untrained young men. Previous studies have shown that functional resistance training yielded a significant positive impact on athletes’ straight-line sprint ability (Yildiz et al., 2019; Keiner et al., 2020). However, inconsistent study findings were also found in trained individuals. For example, Cressey et al. (2007) reported that elite athletes could improve more significantly by performing stable training rather than unstable surface training in 40-yard sprint time, and they can produce better results for other indicators of athletic performance. Given the fact that the present study target is untrained young men, we should exercise caution when interpreting the treatment outcomes. It is noteworthy that untrained individuals adapt more readily, to a great magnitude, and with less need for specificity when performing training under stable or unstable conditions. Gruber and Gollhofer (2004) found that instability resistance training enhanced neuromuscular activation in untrained individuals in the early training phase of muscular action. Similarly, Kibele and Behm (2009) also reported that greater instability could challenge the neuromuscular system to a greater extent than the stable environment in the early stages of resistance training, possibly enhancing strength gains attributed to neuromuscular adaption. However, according to the specificity-of-training principle, training must fit the demands of the task or activity as much as possible, especially for the athletes training in unstable environments (e.g., BOSU ball, Swiss ball or wobble boards) which are not specific to their sporting tasks. More importantly, the effects of early phase training (increased rate of strength development) observed in untrained individuals might not be applicable in cases of trained athletes. Therefore, recent evidence showed that both, stable or unstable training proved valuable in health promotion and physical capacities in untrained individuals, while caution should be duly exercised in applying unstable training to well-trained athletes’ performance and general exercise scenarios.

Some limitations in this study should also be noted. First, this study involved a limited number of performance variables, and it would be imperative to include other additional motor ability tests such as static and dynamic balance, agility tests, and cardiopulmonary fitness in future research, especially in FRT protocol. Secondly, the study participants were limited to young men; thus, the outcomes could not be generalized due to the absence of women or experienced individuals such as athletes. Moreover, the intervention duration was relatively short (6 weeks), which was not enough to cause a significant difference in muscular fitness and physical performance between the two groups. Future studies with large sample sizes and a variety of participants along with longer study periods are required to determine the excellent resistance training pattern for health promotion.

## Conclusion

In summary, there were no differences between 6 weeks of functional resistance training compared to traditional resistance training on upper and lower limbs muscular endurance and performance. Hence, both training patterns were effective methods for strengthening the physique of untrained young men. Nevertheless, given the limitations summarized in this study, it is necessary to be cautious about the study outcome. Furthermore, training on an unstable surface with external load and purposefully challenging the participants’ balance is inherently unsafe. Hence, the coaches, athletes, or amateurs must select appropriate training methods to suit their core strengths for better training results.

## Data Availability

The original contributions presented in the study are included in the article/Supplementary Material, further inquiries can be directed to the corresponding authors.
